# Sensitivity Differences in Fish Offer Near-Infrared Vision as an Adaptable Evolutionary Trait

**DOI:** 10.1371/journal.pone.0064429

**Published:** 2013-05-15

**Authors:** Denis Shcherbakov, Alexandra Knörzer, Svenja Espenhahn, Reinhard Hilbig, Ulrich Haas, Martin Blum

**Affiliations:** Institute of Zoology, University of Hohenheim, Stuttgart, Germany; Monash University, Australia

## Abstract

Near-infrared (NIR) light constitutes an integrated part of solar radiation. The principal ability to sense NIR under laboratory conditions has previously been demonstrated in fish. The availability of NIR in aquatic habitats, and thus its potential use as a cue for distinct behaviors such as orientation and detection of prey, however, depends on physical and environmental parameters. In clear water, blue and green light represents the dominating part of the illumination. In turbid waters, in contrast, the relative content of red and NIR radiation is enhanced, due to increased scattering and absorption of short and middle range wavelengths by suspended particles and dissolved colored materials. We have studied NIR detection thresholds using a phototactic swimming assay in five fish species, which are exposed to different NIR conditions in their natural habitats. Nile and Mozambique tilapia, which inhabit waters with increased turbidity, displayed the highest spectral sensitivity, with thresholds at wavelengths above 930 nm. Zebrafish, guppy and green swordtail, which prefer clearer waters, revealed significantly lower thresholds of spectral sensitivity with 825–845 nm for green swordtail and 845–910 nm for zebrafish and guppy. The present study revealed a clear correlation between NIR sensation thresholds and availability of NIR in the natural habitats, suggesting that NIR vision, as an integral part of the whole spectrum of visual abilities, can serve as an evolutionarily adaptable trait in fish.

## Introduction

Spectral sensitivity of aquatic animals depends on the natural illumination in their habitats [1]. Conditions vary greatly in the different kinds of water bodies. Light impinging surfaces of natural waters encompasses a wide spectral range from ultraviolet (UV) to infrared [2], [3]. In water, however, the spectral band width of solar radiation is considerably reduced. The main factors diminishing the irradiance of underwater light are reflection, scattering and absorption [4]. Attenuation of the incident light due to reflection on the water surface is generally low. The amount of reflection averages around 6.5% [4] and mainly depends on the wavelengths of light, the angle of incidence as well as water wave action. Scattering and absorption of light by water also depends on wavelengths, but decisively on the concentration of dissolved organic materials and the amount of suspended particles [3], [5], [6]. In clear oceanic water blue light of about 470 nm penetrates the most, allowing photopic vision up to depths of 300–500 m [3]. Fish inhabiting this ecosystem show an evolutionary adaptation to the dominating blue wavelengths with a maximal spectral sensitivity of their eyes at 470 nm [3].

Fresh waters are characterized by higher turbidity compared to oceans. This leads to enhanced scattering and absorption of short- and mid-wavelengths spectral components by suspended particles and dissolved organic material [3], [7], [8]. Underwater illumination consequently suffers a shift to longer wavelengths. Freshwater fish have adapted to these conditions and display a corresponding shift in spectral sensitivity of their eyes towards longer wavelengths [1].

In shallow and highly turbid waters long-wave red and near-infrared (NIR) light may even dominate [3]. Spectral sensitivities in the red and near-infrared range may therefore represent evolutionary adaptations of fish species living in such highly turbid habitats. Surprisingly, only little is known about NIR sensation in fish. So far reception of NIR light was shown in a few teleost fish species, namely the common carp *(Cyprinus carpio)* [9], Nile tilapia (*Oreochromis niloticus*) [10]–[12], Mozambique tilapia (*Oreochromis mossambicus)* [13] and *Pelvicachromis taeniatus* [14]. NIR sensation has been shown to be mediated by eyes and not by the pineal organ [9]. Fish use NIR for visual detection of prey [14] and for native untrained phototactic orientation [13]. Increased NIR spectral sensitivity thus may serve as a major factor for evolutionary adaptations to life in shallow fresh water habitats. In the present study we analyzed NIR spectral sensitivity under defined laboratory conditions in fish species, which are exposed to different NIR spectra in their natural habitats. Our results demonstrate a clear correlation between NIR conditions in nature and observed spectral sensitivities under laboratory conditions, strongly suggesting that enhanced NIR spectral sensitivity represents an adaptive trait in freshwater habitats.

## Materials and Methods

### Ethic Statement

All experiments were based on a non-invasive behavioral procedure, which made use of the native swimming behavior of fish. The light intensities used in all experiments did not exceed values observed in natural waters. This study was carried out with approval of the Animal Care Committee of the University of Hohenheim (permission numbers: S287/10 Zo and S310/11 Zo).

### Animals

Five species were used in the present study: guppy *(Poecilia reticulata),* green swordtail (*Xiphophorus hellerii*), zebrafish *(Danio rerio)*, Nile tilapia (*Oreochromis niloticus*) and Mozambique tilapia (*Oreochromis mossambicus*). Guppies (2.5–3 months old, body length 1.2–2.1 cm) were kept at 23–23.5°C, in accordance with their natural requirements [15]. Green swordtail (2–4 months old, 1.3–1.9 cm in length) were kept at 21.5–25.0°C, which is typical for their natural habitats [16]. Zebrafish (6–7 months old, body length 2.9–3.7 cm) were kept at 22.3–24.0°C [17]. Nile tilapia (1–2 months old, body length 1.9–2.6 cm) were kept at 25.0–26.5°C in accordance with natural occurring conditions for this species [18]. Mozambique tilapia (2–3.5 months old, body length 1.8–3 cm) were kept at a temperature of 24.0–26.0°C, which is typical for their natural habitats [19].

Fish were fed daily with TetraMin flakes (Tetra GmbH, Melle, Germany). Tested species were kept at a 12 h/12 h light/dark cycle under artificial illumination (white spectrum fluorescent lamps; Osram, Munich, Germany) with 90% emissions at 400–750 nm. The total irradiance at the water surface was 320 µW/cm^2^ and total photon flux was 9.5*10^14^ photons cm^−2^s^−1^.

### Behavioral Assays

The experimental setup and behavioral testing was as described in detail in Shcherbakov et al. (2012), except that zebrafish were tested in 50 mm Petri dishes, as opposed to the standard 35 mm dishes applied for all other species.

### Behavioral Analysis

Fish behavior was assessed by the custom-made video tracking software BioMotionTrack D.S. [20] using a semi-automated mode. Fish position was determined manually by a mouse click between the eyes, followed by automatic behavioral analysis. Fish behavior was analyzed by calculating the following parameters: (i) swimming time [%] during the experiment, in order to test the motivation of fish for active swimming orientation; (ii) allocation time on the left and right side of the Petri dish with regard to the fish head position [s], to test light dependence of allocation preference; (iii) mean angle [°] of the preferred fish head alignment, with regard to the direction of the NIR light source; (iv) mean angular deviation [°] as a dimension of directional variance; and (v) length of the mean directional vector *R* for each experiment in order to assess a possible significance of directional preference using a Rayleigh test of uniformity [21]–[23]. An *R*-value of 0 indicates uniform distribution in all directions, whereas the maximum possible value *R* = 1 is reached when all vectors point in the same direction. Before performing circular statistical analyses the direction of the NIR-light source (270° for the left-sided experiments and 90° for the right side) was transformed to 0°. All measured angles of preferred directions for each individual were transformed accordingly. For detailed visualization sector diagrams were drawn to show the preferred fish allocation in the Petri dish. Diagrams divided the round swimming vessel into 24 angular sectors of 15 degrees each.

### Statistical Analysis

Calculations of statistical significances for two-sided results (light vs. control side) were preformed using the two-tailed Wilcoxon matched pairs test (Statistica 6.1; StatSoft, Inc., Tulsa, OK, USA). Statistical significance of the length of the mean directional vector *R* was analysed using a Rayleigh test of uniformity [21], [22]. Significance levels were defined as not significant n.s. (*p*≥0.05), *(*p<*0.05), **(*p<*0.01), and ***(*p*<0.001).

### Spectral Measurements and Behavioral Experiments

**Figure 1:**
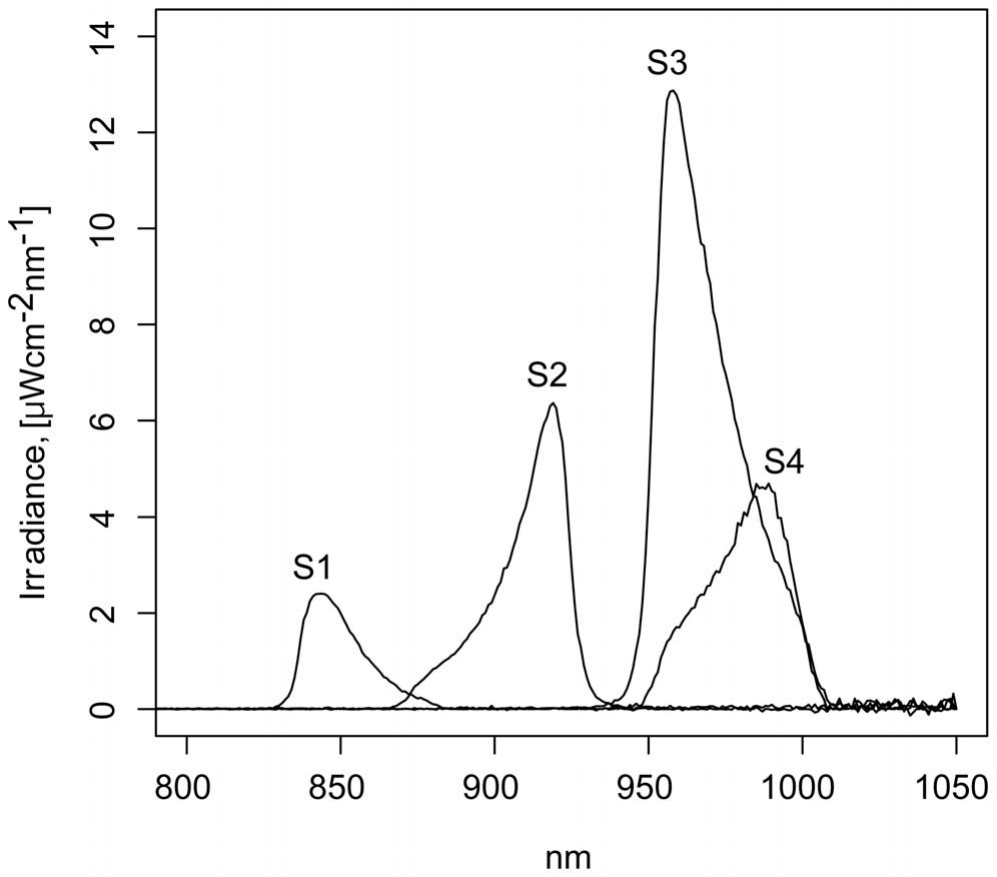
Spectra used. The range of each spectrum was measured by spectroradiometer ILT950 at a distance of 10 cm between swimming vessel and light source. S1: 825–890 nm; S2: 845–950 nm; S3: 910–1020 nm; S4: 930–1020 nm. For wavelengths above 1020 nm only noise of the detecting device was observed.

Spectral measurements of light sources were carried out at the position of the swimming vessel using an ILT 950 spectroradiometer (International Light Technologies, Peabody, MA, USA). The following four spectra were used: (S1) near-infrared radiation emitted by an LEDs-based NIR-light source with wavelengths corresponding to a maximum intensity of λ_(max) = _845 nm (Conrad Electronic, Hirschau, Germany). Use of band pass filter D860/40X (Chroma Technology Corp., Bellows Falls, VT, USA) narrowed the measurable spectrum of NIR light to a range of 825–890 nm (λ_(max)_ = 845 nm; Figure 1 and Table 1); (S2) NIR light source LED L-53F3C (Kingbright Elec. Co., Ltd., Taipei, Taiwan), with λ_(max)_ = 940 nm and full width at half maximum (FWHM) = 50 nm. Band pass filter D900/50X (Chroma Technology Corp., Bellows Falls, VT, USA) reduced the emitted spectrum to 845–950 nm and shifted λ_(max)_ to 920 nm (Figure 1 and Table 1); (S3) LED L-53F3C with the band pass filter Z980/50X (Chroma Technology Corp., Bellows Falls, VT, USA), which narrowed the spectrum to 910–1020 nm (λ_(max)_ = 960 nm; Figure 1 and Table 1); (S4) NIR-light source LED 980-03 (Roithner Lasertechnik GmbH, A-1040 Wien, Austria) with λ_(max)_ = 985 nm and FWHM = 45 nm. Band pass filter Z980/50X (Chroma Technology Corp., Bellows Falls, VT, USA) resulted in emission at 930–1020 nm (λ_(max)_ = 986 nm; Figure 1 and Table 1).

**Table 1:** Spectral characteristics of applied light.

Spectrum identifier	λ_(max)_ (nm)	Irradiance_max_ (µWcm^−2^nm^−1^)	Total photon flux, (10^14^ photons cm^−2^s^−1^)	Measurable spectral range (nm)
S1	845	2.39	2.4	825–890
S2	920	6.26	7.2	845–950
S3	960	12.87	18.2	910–1020
S4	986	4.69	7.4	930–1020

To assess possible confounding effects of thermal radiation emitted from the applied light sources, 30 temperature measurements were performed for one experimental series of each spectrum, using an infrared thermometer (VOLTCRAFT IR-230; Conrad Electronic, Hirschau, Germany). Temperatures were measured at the surface of the activated NIR light source, and at the surface of the control light source in ‘off’ position (Table 2).

**Table 2:** Mean surface temperature of emitting (NIR) and non-emitting (control) light sources.

Spectrum identifier	Spectral range (nm)	Temperature of the emitting (NIR) light sources, °C	Temperature of the not emitting (control) light sources, °C	Temperature difference, °C
S1	825–890	26.1±0.2	25.7±0.3	0.4
S2	845–950	26.7±1.1	25.7±1.1	1.0
S3	910–1020	27.9±0.2	27.4±0.3	0.5
S4	930–1020	26.9±0.5	25.9±0.0	1.0

Thirty measurements were carried out with 30 fish for each spectrum.

## Results and Discussion

In order for NIR spectral sensitivity to qualify as an adaptive trait, the following predictions should be fulfilled: (i) Thresholds for NIR-sensation and, consequently, NIR-based phototactic orientation should vary between species; (ii) NIR sensitivity of species from habitats with increased turbidity should be higher than that of fish from waters with higher transparency; (iii) Spectral sensitivities should be based on photo- and not on thermoreception; (iv) NIR spectral sensitivities of species should correlate with the physical characteristics of NIR light penetration of water columns in their natural habitats.

These hypotheses were experimentally tested using the phototactic swimming assay described previously [13]. Typically, fish spent >87% of experimental time in locomotion (Table 3), revealing an active orientation swimming behavior under all spectra applied. The lowest level of swimming activity with the highest variance between individuals was observed in Mozambique tilapia (Table 3).

**Table 3:** Mean swimming time (%) of fish during experimental testing period.

Spectrum identifier	Spectral range (nm)	Green swordtail	Guppy	Zebrafish	Nile tilapia	Mozambique tilapia
S1	825–890	97.8±3.2	99.0±0.5	98.5±2.3	97.6±1.7	89.0±20.9
S2	845–950	99.0±0.3	99.1±0.4	99.1±0.3	98.3±1.0	87.1±19.4
S3	910–1020	98.9±0.5	98.9±0.4	97.1±10.3	97.7±5.6	91.4±20.4
S4	930–1020	–	–	–	97.4±4.5	99.2±0.4

### Fish Species are Sensitive to NIR of Different Spectral Range

**Figure 2:**
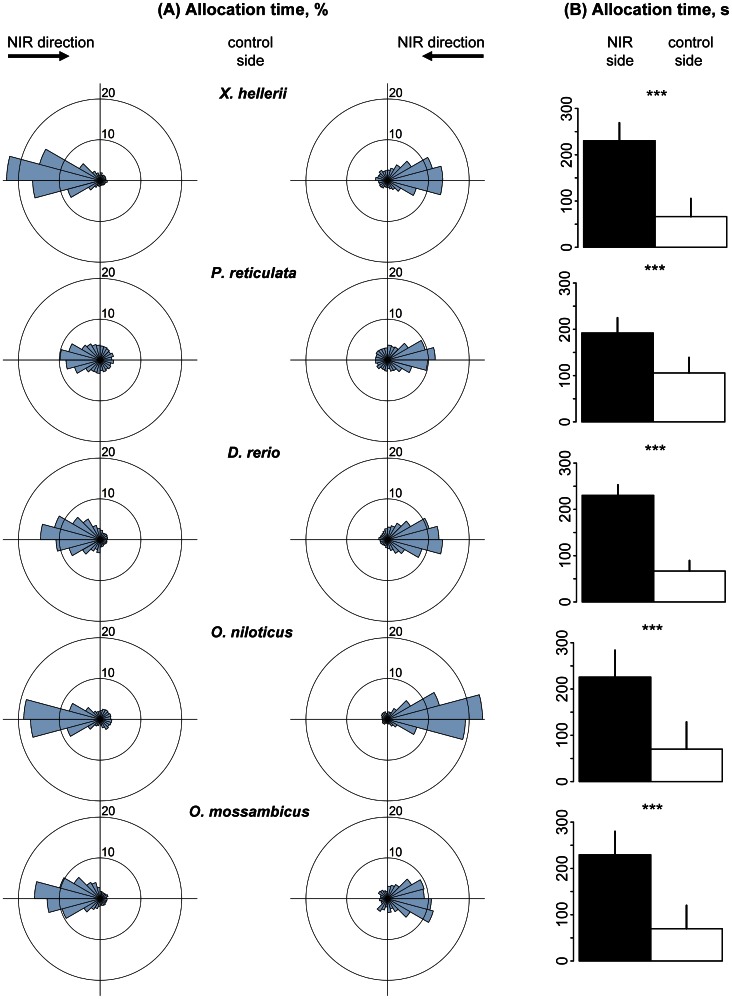
Allocation preference of fish in the spectral range between 825 and 890 nm (Spectrum S1). (A). Sector diagrams show mean allocation time [%] with regard to mean head position of fish in 24 sectors of the swimming vessel, representing 15° each. Each left and right sector diagram shows mean data for 15 fish of each species. (B). Bar graphs show mean allocation time of individuals of each fish species [s] ± standard deviation (n = 30) for NIR (black) and control halves (white) of the swimming vessel, respectively.

In the spectral range between 825 and 890 nm all tested species spent significantly more time in the NIR half compared to the control side (Figure 2). Allocation times on the NIR side were significantly higher than on the control side in all cases (*p*<0.001), with factors of 3.5 for swordtails, 1.8 for guppies, 3.4 for zebrafish, 3.2 for Nile and 3.3 for Mozambique tilapia (Figure 2). All tested species showed significant head alignment preferences towards the NIR light sources (Tables 4 and 5). The mean directional vector *R* between the middle of the Petri dish and the mean head positions reached values >0.74 (Table 5).

**Table 4:** Mean angle (°) of the preferred fish head alignment with regard to direction of the NIR-light source (± mean angular deviation).

Spectrum identifier	Spectral range (nm)	Green swordtail	Guppy	Zebrafish	Nile tilapia	Mozambique tilapia
S1	825–890	359.8±12.9	358.7±15.1	1.7±9.8	356.2±25.0	4.6±20.8
S2	845–950	16.8±47.4	12.6±43.3	0.0±45.9	358.9±19.5	7.6±25.4
S3	910–1020	330.8±44.8	210.8±46.9	348.0±47.2	340.9±30.7	342.5±36.7
S4	930–1020	–	–	–	347.1±36.5	27.5±40.7

Direction to NIR-light source = 0°.

**Table 5:** Length of the mean directional vector *R*.

Spectrum identifier	Spectral range (nm)	Green swordtail	Guppy	Zebrafish	Nile tilapia	Mozambique tilapia
S1	825–890	0.93***	0.91***	0.96***	0.75***	0.83***
S2	845–950	0.10 n.s.	0.25 n.s.	0.16 n.s.	0.85***	0.74***
S3	910–1020	0.20 n.s.	0.12 n.s.	0.11 n.s.	0.62***	0.46**
S4	930–1020	–	–	–	0.47**	0.34*

**Figure 3:**
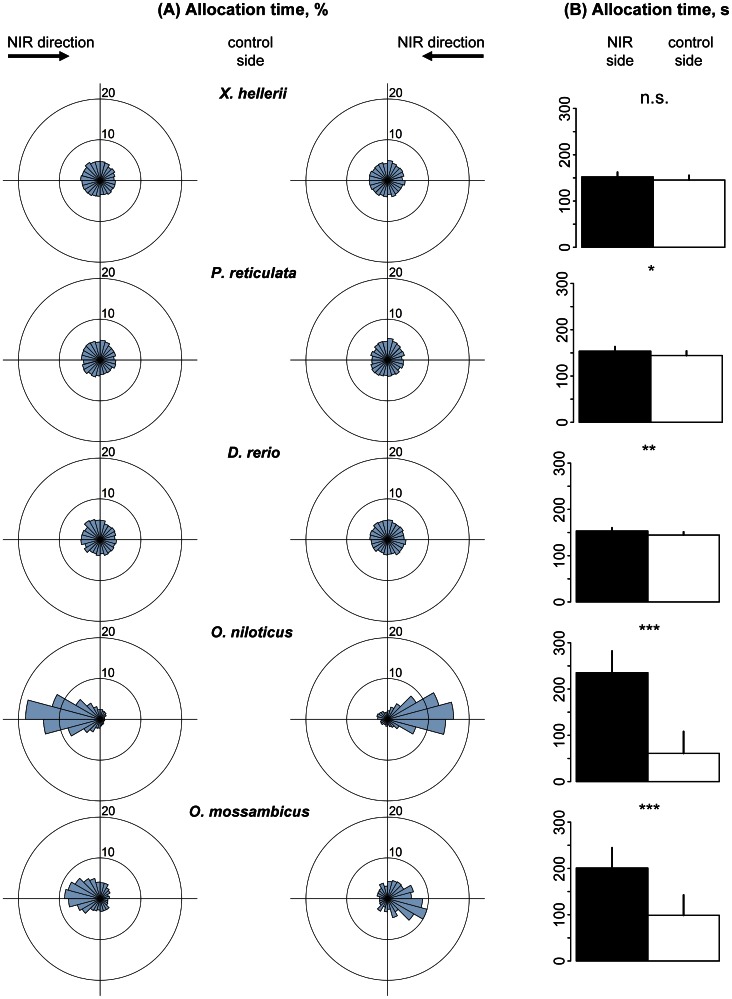
Allocation preference of fish in the spectral range between 845 and 950 nm (Spectrum S2). (A). Sector diagrams show mean allocation time [%] with regard to mean head position of fish in 24 sectors of the swimming vessel, representing 15° each. Each left and right sector diagram shows mean data for 15 fish of each species. (B). Bar graphs show mean allocation time of individuals of each fish species [s] ± standard deviation (n = 30) for NIR (black) and control halves (white) of the swimming vessel, respectively.

When the spectrum was shifted to 845–950 nm, Nile and Mozambique tilapia spent 3.9 and 2.0 times more time, respectively, in the NIR half of the swimming vessel compared to the control side (Figure 3, p<0.001). Both revealed a significant preference to align their heads into the direction of the NIR light source, with *R*>0.73 (Tables 4–5, P<0.001). The other three tested fish species, however, showed considerably different results (Figure 3). Allocation time of zebrafish and guppies were still 1.06 times higher in the NIR compartment compared to the control side. Significances, however, were much lower (zebrafish: *p*<0.01; guppy: *p*<0.05). In Green swordtails, no significant differences between test and control side were found, indicating the absence of NIR vision at this spectral range (Figure 3). Head alignment preferences showed no significances in these three species when circular statistics were applied (*R*<0.26; Tables 4–5).

**Figure 4:**
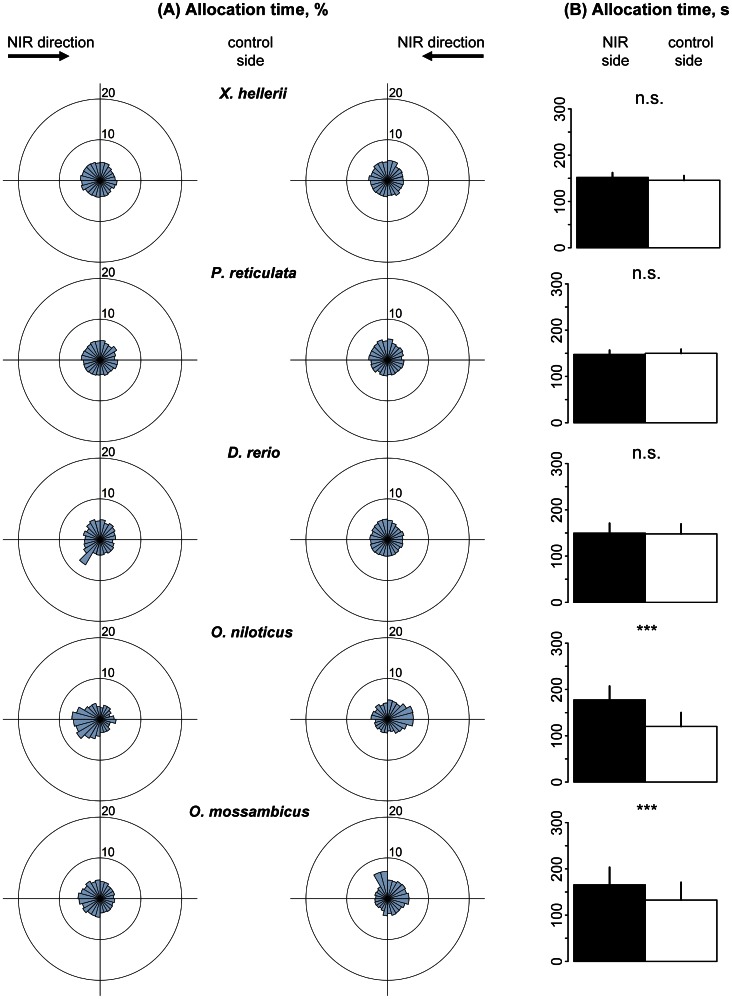
Allocation preference of fish in the spectral range between 910 and 1020 nm (Spectrum S3). (A). Sector diagrams show mean allocation time [%] with regard to mean head position of fish in 24 sectors of the swimming vessel, representing 15° each. Each left and right sector diagram shows mean data for 15 fish of each species. (B). Bar graphs show mean allocation time of individuals of each fish species [s] ± standard deviation (n = 30) for NIR (black) and control halves (white) of the swimming vessel, respectively.

This decrease in NIR spectral sensitivity was more pronounced when the spectrum was further shifted to the long wave range (910–1020 nm). Nile and Mozambique tilapia still spent significantly more time in the NIR half of the swimming vessel compared to the control side (Figure 4; *p*<0.001). The mean directional vector *R* reached 0.62 in Nile (p<0.001) and 0.46 in Mozambique tilapia (p<0.01; Table 5). The other three species, however, did not reveal any NIR spectral sensitivity under these conditions (Figure 4, Tables 4–5).

**Figure 5:**
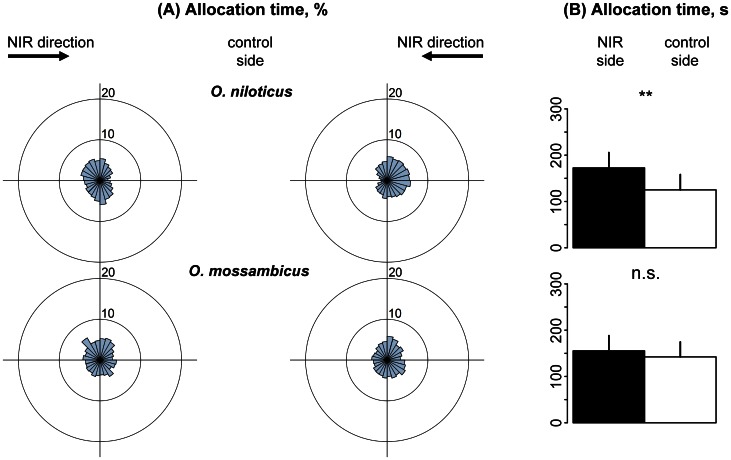
Allocation preference of fish in the spectral range between 930 and 1020 nm (Spectrum S4). (A). Sector diagrams show mean allocation time [%] with regard to mean head position of fish in 24 sectors of the swimming vessel, representing 15° each. Each left and right sector diagram shows mean data for 15 fish of each species. (B). Bar graphs show mean allocation time of individuals of each fish species [s] ± standard deviation (n = 30) for NIR (black) and control halves (white) of the swimming vessel, respectively.

A further shift to even longer wavelengths was therefore only investigated in Nile and Mozambique tilapia. When fish were tested under NIR light of 930–1020 nm (Figure 5) Nile tilapia spent 1.4 times more time in the NIR half of the swimming vessel compared to the control side (*p*<0.01). Mozambique tilapia, in contrast, revealed no significant differences in allocation time between NIR and control halves (*p* = 0.1; Figure 5). Both species, however, showed a significant preference to align their heads into the direction of the NIR light source with *R* = 0.47 for Nile tilapia and *R* = 0.34 for Mozambique tilapia (p<0.01 and p<0.05, respectively; Tables 4–5).

**Figure 6:**
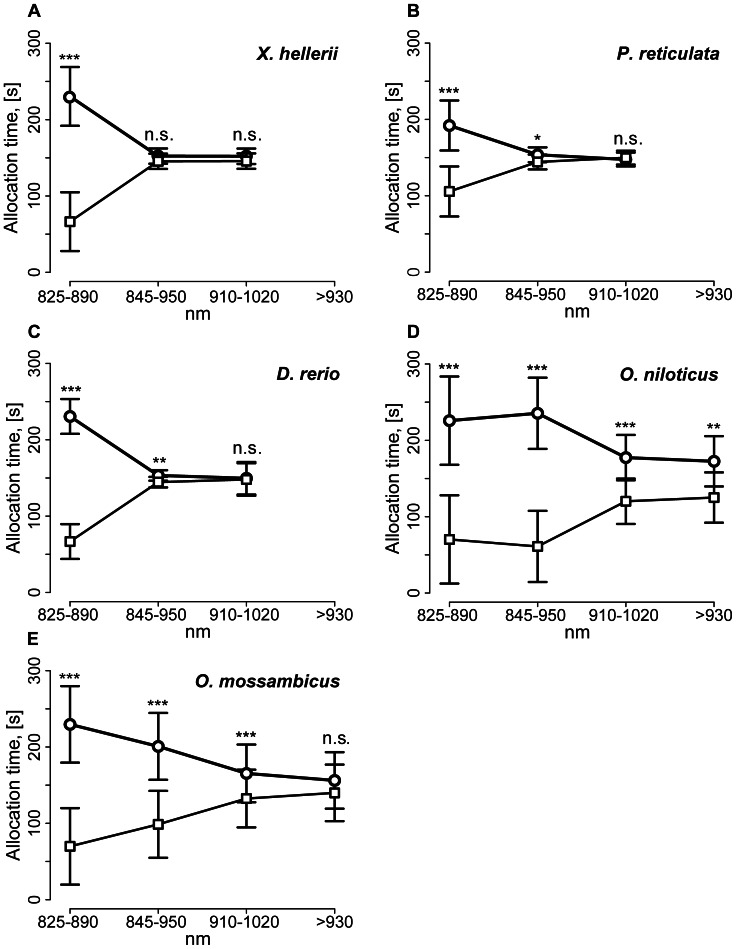
Comparison of allocation preference of tested species in different NIR spectra. (A–E) Lines represent mean allocation time [s] ± standard deviation in NIR (line with circles) and control (line with squares) halves of the swimming vessel for each tested spectrum and species.

NIR vision thus was detectable in all five fish species analyzed here. Detection of NIR therefore might be a general characteristic of freshwater fish. Clear differences, however, were found with respect to spectral sensitivities in the NIR range. Nile and Mozambique tilapia were species with increased spectral sensitivity to NIR light up to >930 nm (Table 5). Zebrafish and guppy revealed light dependent behavioral reactions at a range of 845–910 nm, while green swordtail reacted only at 825–845 nm (Figure 6). As each spectrum applied represented wavelength distributions of different intensities, more precise range determinations of spectral sensitivity thresholds could not be derived from these results. Remarkably, total photon flux of spectrum S3 (910–1020 nm) was about 8 times higher than that of S1 (825–890 nm). Under S1 conditions, however, all fish responded to NIR light, whereas only Nile and Mozambique tilapia revealed phototactic reactions to NIR under S3 illumination. These data suggest that the failure of zebrafish, guppy and sword tail to respond to S3 light was not due to insufficient photon density, but to a lack of photoreceptor spectral sensitivity in the >910 nm wavelength range. Alternatively, photoreceptors with sensitivities >910 nm might be present but underrepresented in zebrafish, guppy and sword tail to result in lower absolute sensitivity.

### NIR Sensitivities Correlate with Turbidity of Water in Natural Habitats

The present results allow the classification of tested species into two clearly separated sensitivity groups: Nile and Mozambique tilapia revealed high NIR sensitivities, while zebrafish, guppy and green swordtail were characterized by low NIR spectral sensitivities. The observed differences between species may be the result of evolutionary adaptation to the prevailing illumination conditions in the various habitats. Indeed, zebrafish prefer waters of relatively high transparency [17], while guppies and green swordtail typically inhabit clear streams [24]–[26]. Low NIR spectral sensitivity therefore correlates with a preference for clear, i.e. highly transparent aquatic habitats. Nile tilapia, on the other hand, inhabits marginal waters and floodplain pools with dominating middle and long-wave spectral regions [27], and Mozambique tilapia prefers even turbid waters [28]. These two tilapia species are closely related and share many aspects of their basic biology. Both are widely used in aquaculture where production success is based particularly on their ability to survive and to reproduce in waters of poor quality [18], [29], [30]. Under such conditions, considerable amounts of suspended particles and dissolved organic materials may lead to an increased scattering of short and middle wavelengths of light. The relative proportion of red and NIR illumination may therefore rise considerably [3], [7], [8]. In summary, the observed sensitivity differences in the NIR range correlate well with preferences for distinct water qualities in the five fish species tested here.

### NIR Sensation is Based on Photoreception

When assessing long wavelength radiation sensitivities, thermal sensitivity may represent a major confounding factor [14]. In order to investigate the contribution of the NIR sources applied here, temperatures were measured at NIR and control light sources at the different settings (S1–S4; cf. Material and Methods). Thermal measurements revealed a slight increase in the mean temperature from 26.1–27.9°C at the surface of emitting light sources (Table 2), compared to 25.7–27.4°C on the surface of the control side. According to Wien’s displacement law, thermal sources with temperatures of 26.1–27.9°C emit most of their radiation at wavelengths of about 9.6–9.7 µm, which can be sensed by thermo- but not by photoreceptors [14]. A comparison of temperature and behavioral data strongly argues for photoreception as opposed to thermoreception, however, as differences were marginal with a maximum of 1°C (Table 2), and longer wavelength, i.e. a potentially higher percentage of thermal radiation, resulted in progressive loss of phototactic swimming behavior in 3/5 species. We can therefore exclude any influence of thermal radiation on allocation preferences in the five species tested here. The observed orientation in fish thus was based on the reception of light in the near-infrared spectrum and not on the detection of thermal radiation, confirming our previous results on Mozambique tilapia [13].

### NIR Sensitivities Correlate with Physical Characteristics of NIR Light Penetration in Natural Habitats

Our hypothesis that observed differences in NIR sensation represent evolutionary adaptations to light penetration in different ecosystems predicts that physical parameters of NIR penetration in aquatic habitats correlate with the observed sensitivities. In particular, the question arises whether fish may even sense longer wavelength NIR than the most sensitive species investigated here, i.e. Nile and Mozambique tilapia, with an NIR spectral sensitivity above 930 nm. The reduced statistical significance in this spectral range, however, suggests that the spectral sensitivity limit for this species should be not much higher than 930 nm.

What significance might this high NIR spectral sensitivity have for that particular fish species? A model calculation of light penetration in clear water may help to uncover the potential ecological relevance of our data. As outlined above, reflection, scattering and absorption define the intensity of light penetration in water. The level of reflection is usually lower than 14% [31]–[35]. The degree of absorption and scattering depends on wavelengths of penetrating light and on the concentration of suspended particles and dissolved colored materials in the water column, and may differ considerably between different natural water bodies. Absorption of light by pure water mainly depends on the wavelengths [5], [6], [36]. Light scattering of pure water is low and can be neglected. Using natural absorption coefficients [37] it is possible to compare light penetration of different wavelengths in pure water, which represents a standardized medium, without the need to consider variable other conditions, which occur in different natural water bodies. For our calculation we used the absorption law of Lambert-Beer:
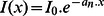
with:

*I_0_*: Incident (primary) light intensity;

*I(x):* Intensity of light after penetrating a layer of pure water of thickness x;

*a_n_*: (natural) absorption coefficient;

*x:* thickness of water layer.

**Figure 7:**
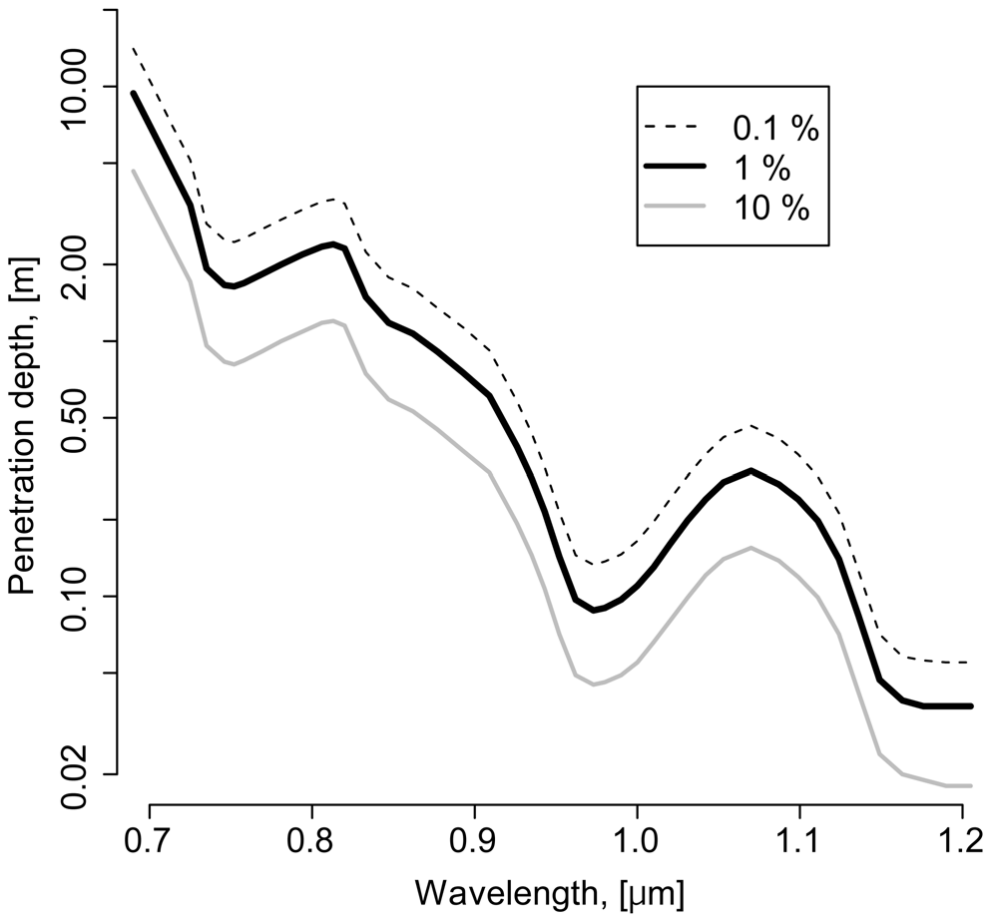
Penetration depths of light at spectral ranges of 0.7–1.2 µm. Curves represent wavelength-dependent penetration of fractions of 10% (gray line), 1% (black line) and 0.1% (black dashed line) of initial light intensities. Graph is based on data from Palmer and Williams [37].

Our calculation demonstrates that in pure water a reduction of the primary light intensity I_0_ by a factor of 100 will be reached at progressively shallower depths when the wavelengths increase: for wavelengths of 702 nm at a depth of 6.6 m, for 752 nm at 1.6 m, for 806 nm at 2.3 m, for 847 nm at 1.2 m, for 909 nm at 61 cm, for 952 nm at 14 cm, and for 1000 nm at 11 cm. A detailed depiction of penetration depths of light in the spectral range of 0.7–1.2 µm is presented in Figure 7. Light of wavelengths >930 nm may penetrate only a few cm, even in pure water. A spectral sensitivity threshold of 930–950 nm may thus represent a natural biological and physical limit for NIR spectral sensitivity for any aquatic organisms. The observed reduction of NIR spectral sensitivity above 930 in the most sensitive species Mozambique and Nile tilapia correlates well with physical characteristics of NIR light penetration in water.

Although the NIR light is available in depths up to about 2 m in the water column, green swordtail, guppy, zebrafish, Nile and Mozambique tilapia inhabit surface waters. NIR is therefore an integral part of the visual spectrum in the natural habitats of these species. Moreover, high far red and NIR sensitivities may be especially relevant for Nile and Mozambique tilapia, which inhabit shallow waters with increased turbidity and thus an increased relative proportion of red and near-infrared illumination compared to clear waters.

### Evolution of NIR Sensitivity

Which organs, cells and molecules do we have to consider when discussing NIR vision as an adaptive trait? Our previous study [13] and results presented here revealed that fish were able to sense NIR by photoreception and not by thermoreception. Other work had shown that Nile tilapia perceive NIR through their eyes and not through the pineal organ [9]. Furthermore, the ability to use NIR light for vision was recently demonstrated in Nile tilapia and *Pelvicachromis taeniatus* [11], [14]. A possible reception of NIR light in deeper regions of the brain or in the pineal organ would additionally require efficient penetration of light through tissue, which most certainly would be much attenuated, further reducing the likelihood for infrared sensitivity of these organs [38]. Together these findings and arguments suggest that the observed swimming orientation was dependent on eye-based NIR-vision.

In Nile tilapia violet-sensitive, short-, medium- and long-wavelength sensitive cones were found, with the highest sensitivities at longer wavelengths [27]. It was suggested that both types of photoreceptors, cones and rods, were sensitive to NIR [9]. The highest spectral sensitivity of rods usually occurs in the shorter wave spectral range (compared to the long-wavelength sensitive cones) and reveals typically peaks of absorption and sensitivities below 540 nm [39]–[45], reducing the probability of rod participation in NIR sensation. Besides these receptors several other forms of photoreceptors were described in fish, e.g. melanopsin and vertebrate ancient (VA) opsin based photoreceptors [38], [46], [47]. As the maximal sensitivity of these photopigments was found in rather short wave regions [44], [48], they should not be involved in the infrared sensation in fish. Long wavelength sensitive cones, on the other hand, represent the most red sensitive photoreceptor type in fish eyes [43]. They may therefore be the most likely candidates for the observed NIR-sensation.

Remarkably, big differences in relative expression levels of retinal opsin genes were reported in African cichlids, with an about 80% proportion of long wavelength (LWS) opsin in Nile tilapia [49]. In zebrafish the relative expression of LWS cone opsin genes was considerably lower than that of short wavelength (SWS) ones [50]. These published data on relative opsin gene expression levels correlate well with the differences in NIR vision observed in this study. It will be interesting to study opsin gene expression levels in the other three species tested here, in order to confirm a possible functional relevance of opsin gene expression in the context of NIR vision.

NIR-sensation is an integrated part of the whole spectral sensitivity in fish. Although in waters of high turbidity the relative part of red and infrared light increases, shorter wavelengths are available under such environmental conditions as well [4]. Therefore, a possible biological role of NIR-sensation has to be considered in the context of the whole spectral sensitivity. An increased red and near infrared sensitivity may improve visual abilities in species from turbid aquatic habitats, i.e. when illumination shifts to longer wavelengths. This long wave sensory shift may be of biological relevance for those aspects of life cycles in fish which depend on visual abilities, i.e. foraging behavior [14], orientation [13], predator avoidance, intraspecific communication [7], [51]–[54] and sexual selection [7].
